# Bariatric Surgery for Morbid Obesity: Tehran Obesity Treatment Study (TOTS) Rationale and Study Design

**DOI:** 10.2196/resprot.5214

**Published:** 2016-01-20

**Authors:** Maryam Barzin, Farhad Hosseinpanah, Mohammad Ali Motamedi, Parvin Shapoori, Peyman Arian, Maryam Alsadat Daneshpour, Golale Asghari, Ahmad Teymoornejad, Ali Eslamifar, Davood Khalili, Behzad Jodeiri, Shahram Alamdari, Fereidoun Azizi, Alireza Khalaj

**Affiliations:** ^1^ Obesity Research Center Research Institute for Endocrine Sciences Shahid Beheshti University of Medical Sciences Tehran Islamic Republic of Iran; ^2^ A Minimally Invasive Surgery Research Center Rasoul-e-Akram Hospital Iran University of Medical Sciences Tehran Islamic Republic of Iran; ^3^ Obesity Treatment Center Faculty of Medicine Shahed University Tehran Islamic Republic of Iran; ^4^ Cellular and Molecular Endocrine Research Center Research Institute for Endocrine Sciences Shahid Beheshti University of Medical Sciences Tehran Islamic Republic of Iran; ^5^ Nutrition and Endocrine Research Center Research Institute for Endocrine Sciences Shahid Beheshti University of Medical Sciences Tehran Islamic Republic of Iran; ^6^ Department of Clinical Research Pasteur Institute of Iran Tehran Islamic Republic of Iran; ^7^ Prevention of Metabolic Disorders Research Center Research Institute for Endocrine Sciences Shahid Beheshti University of Medical Sciences Tehran Islamic Republic of Iran; ^8^ Obesity Research Center Reaserch Institute for Endocrine Sciences Shahid Beheshti University of Medical Sciences Tehran Islamic Republic of Iran; ^9^ Endocrine Research Center Research Institute for Endocrine Sciences Shahid Beheshti University of Medical Sciences Tehran Islamic Republic of Iran; ^10^ Obesity Treatment Center Department of Surgery Shahed University Tehran Islamic Republic of Iran

**Keywords:** obesity, overweight, weight loss, bariatric surgery

## Abstract

**Background:**

Obesity is a major health concern in the Middle East and worldwide. It is among the leading causes of morbidity, mortality, health care utilization, and costs. With bariatric surgery proving to be a more effective treatment option for overweight and obesity, the need for systematic assessment of different procedures and their outcomes becomes necessary. These procedures have not yet been described in detail in our region.

**Objective:**

We aim to undertake a prospective study evaluating and comparing several surgical bariatric procedures in an Iranian population of morbid obese patients presenting to a specialized bariatric center.

**Methods:**

In order to facilitate and accelerate understanding of obesity and its complications, the Tehran Obesity Treatment Study (TOTS) was planned and developed. This study is a longitudinal prospective cohort study in consecutive patients undergoing bariatric surgery. TOTS investigators use standardized definitions, high-fidelity data collection system, and validated instruments to gather data preoperatively, at the time of surgery, postoperatively, and in longer-term follow-up.

**Results:**

This study has recruited 1050 participants as of September 2015 and is ongoing.

**Conclusions:**

This study will ensure creation of high-level evidence to enable clinicians to make meaningful evidence-based decisions for patient evaluation, selection for surgery, and follow-up care.

## Introduction

### Health Burden of Severe Obesity

Obesity is a growing health concern in Iran and is now a global pandemic. The latest World Health Organization (WHO) report on obesity indicates that the overweight and obese population is growing. In fact, the prevalence of obesity has nearly doubled worldwide since 1980, and more than 10% of the world’s adult population is obese [1]. Although the obesity prevalence remained unchanged during the last 10 years in the United States [2], it is still high: 35.5% of the adult population in the United States is obese, which is defined as a body mass index (BMI) ≥30, 15.5% have a BMI over 35, and 6.3% are morbidly obese (BMI ≥40) [3]. Obesity prevalence is on the rise in developing countries due to demographic, socioeconomic, and nutritional transitions [4], and Iran is no exception: 10.8% and 3.4% of the population are obese and morbidly obese, respectively [5].

Severe obesity is associated with comorbidities such as type 2 diabetes mellitus, hypertension, cardiovascular disease, degenerative joint disease, and sleep apnea [6,7]. It has a major impact on quality of life [8] and psychosocial health as well, and major depression is seen in 7% of this population [9].

### Treatment of Severe Obesity

Over the past few decades, there has been a major change in trends of obesity treatment. Lifestyle modifications can, at best, induce a 5-10% weight loss and improve obesity-related morbidities to a limited extent [10]. However, advancements of bariatric surgery in less invasive and safer techniques, along with the evidence-supported superior results over lifestyle modifications, rendered surgery a better treatment option [11-13]. A recent meta-analysis found a 26 kg weight difference between surgical versus non-surgical treatment of morbidly obese patients in 1-2 years follow-up [14]. A Utah obesity study found this difference at 27% of initial body weight between the two groups after 6 years [15]. Results of the Swedish Obese Subjects study after 20 years of follow-up showed 18% mean weight loss for the surgical group, compared with 1% for the non-surgical [16]. Another meta-analysis including 161,756 patients showed BMI loss of 12-17 kg/m^2^ after 5 years following bariatric surgery [17]. Overall, evidence now supports the choice of bariatric surgery over lifestyle changes for the treatment of severe obesity not only because of excess weight loss (EWL) reductions, but also because of significant benefits in terms of comorbidities and prolonged survival [13,18,19]. However, surgery is not without risks; perioperative mortality is estimated about 0.3%, and 30-day complication rate about 4.1% [20].

### Results of Bariatric Surgical Procedures

There are various surgical procedures for the treatment of severe obesity. Roux-en-Y gastric bypass (RYGB), laparoscopic adjustable gastric banding (LAGB), laparoscopic sleeve gastrectomy (LSG), and biliopancreatic diversion with or without duodenal switch (BPD/BPD-DS) are the recommended and most commonly performed today [21]. Laparoscopic procedures are in general preferred over the open approach [11]. Newer techniques such as plication and mini-gastric or single loop gastric bypass are also emerging. Besides the variable effectiveness across procedures, each has risks and benefits.

In terms of effectiveness, since no single study has incorporated all the techniques, inference must be made through pooled data analyses. In general, BPD/BPD-DS results in more EWL (especially for patients with a very high BMI). RYGB and LSG have shown somewhat comparable results, although some studies have shown a significant advantage for RYGB [22]. LAGB comes after these techniques. Their effect on improvement of comorbidities follows the same order [23,24].

In terms of complication rate and safety, some studies have shown a significant difference between procedures. A meta-analysis of 85,048 patients found a 30-day mortality of 1.11% for BPD-DS, 0.16% for RYGB, and 0.06% for AGB [25]. Another study evaluating LSG found its rate between LAGB and RYGB to be 0.11%, 0.05%, and 0.14% respectively [22]. Overall complication rate is estimated between 4-25% [26]. Most common adverse effects after bariatric surgery are iron deficiency anemia after bypass operations (15%) and reoperations (8%) [14]. RYGB, among others, is associated with higher complication rates, while LAGB showed more reoperation rates, up to 35% according to a large cohort study [27].

There is still no universal agreement to recommend one procedure over another. Choice of the procedure depends on many factors such as the available expertise, risk stratification, patient preferences, and goal of therapy (weight loss vs glycemic control) [25].

### Knowledge Gap

We aim to undertake a prospective study evaluating and comparing several surgical bariatric procedures in an Iranian population of morbidly obese patients presenting to a specialized bariatric center. As newer techniques such as LSG (as a stand-alone operation) and mini-gastric bypass are emerging and gaining popularity, their role in bariatric surgery needs to be evaluated before incorporation into general practice.

This is one of the very first studies of its kind in the Middle East and addresses the knowledge gap on the effectiveness, safety, and efficacy of bariatric surgical techniques, including LSG, mini-bypass, and gastric plication [28].

## Methods

### Objectives and Study Variables

The main objectives of the study are to identify perioperative problems of morbidly obese patients; to assess and compare the effectiveness of different bariatric surgical techniques on metabolic syndrome and other obesity-related comorbidity, by measuring anthropometric indices, EWL rates, glucose homeostasis, blood pressure, lipid profile, hormone levels, and nutritional status; to assess the psychosocial aspects of obesity before and after the bariatric surgery, and long term, including quality of life and depression; and to explore the mechanisms and underlying pathophysiology in the field of obesity and its comorbidities through pathological, genetic, and molecular studies. The study variables are summarized in Table 1.

### Overall Study Design

Tehran Obesity Treatment Study (TOTS) is an ongoing, single-institution, prospective study commencing March 2013. The TOTS enrolls patients to undergo a bariatric procedure based on an individualized clinical decision plan. It is organized into 4 phases: preoperative evaluation, operation, short-term follow-up, and long-term follow-up.

#### Preoperative Evaluation

Baseline data collected by the research team includes demographic data, anthropometric indices, physical examination, quality of life score, psychological data, and physical activity levels. Patients are then referred for several assessments including cardiac and respiratory (including ECG, echocardiogram, chest x-ray, pulmonary function test, and/or polysomnography), gastrointestinal (endoscopy and/or barium meal and abdominal ultrasound), endocrine, and psychological assessments. Patient participation depends on approval by all the consultants. Blood and urine samples are collected preoperatively (see Multimedia Appendix 1). An obesity expert physician then assesses each individual’s data. Written informed consent is obtained from all the participants, including minors and adolescents who are told fully about the study. After their approval for participation, written consent is obtained from their parents or guardians.

#### Operation

Patients undergo one of several different bariatric procedures. Detailed data regarding anesthesiology, operation, and recovery are collected.

#### Short-Term Follow-Up

Post-operative follow-up for complications of surgery as well as other issues are sought and documented. Data regarding anthropometric indices and physical examination are collected at 1 month and 3 months after surgery. At the next visit 6 months postoperatively, a more detailed assessment, including blood samples, is done.

#### Long-Term Follow-Up

Patients are followed annually and reassessed on all baseline variables.

### Subject Recruitment and Eligibility For Surgery

Severely obese patients presenting to the Tehran Obesity Treatment Center are examined by an obesity expert in the clinic to evaluate whether they meet the study inclusion criteria. Patients then attend a free-of-charge monthly comprehensive seminar in order to increase their awareness on the subject and promote active participation in the treatment plan. After providing written informed consent, patients proceed with individualized comprehensive sessions and decisions about suitability of surgery and the specific technique are made.

Inclusion criteria were 15-65 years old, BMI levels ≥40 kg/m^2^ or 30<BMI<35 kg/m^2^ with a medical comorbidity/failure of intensive medical treatment for at least 1 year, acceptable surgical risk, and able and willing to provide informed consent and assure regular follow-up.

Exclusion criteria were obesity due to a treatable medical disease (eg, endocrine abnormality); any other medical, psychological, or social condition which, in the opinion of the investigators, would interfere with safe completion of the study protocol; high operative risk; contraindication to bariatric surgery or weight loss; active drug addiction; nursing, pregnant, or intending to become pregnant in the following year; and unable or unwilling to complete questionnaires or expected to experience difficulty with attendance of visits or completion of study.

### Surgical Procedures

Bariatric procedures that are performed in this study include RYGB, LAGB, LSG, and mini-gastric bypass. Newer techniques such as gastric plication are also considered as a treatment option in suitable candidates. In order to record all aspects of the procedures from pre-op to postop, we broke down each of the surgical procedures into its components (eg, length of alimentary limb, pouch size) and structured a measuring scheme.

A single surgical team will perform all operations under general anesthesia, with the patient in the supine position. A standard 5-port laparoscopic technique with the bed in the reverse Trendelenburg position is used. Patients not suitable for laparoscopy will undergo a traditional laparotomy (see Multimedia Appendix 1).

**Table 1:** TOTS variables and their assessment.

Items^a^		Baseline	Discharge	1 month	3 months	6 months	12 months	Annually
**Demographic & medical history** ^b^								
	Date of birth, sex, income, insurance	✓						
	Marriage & education	✓						
	Past medical history	✓						
	Family history	✓						
	Medications	✓	✓	✓	✓	✓	✓	✓
**Behavioral & psychosocial assessment**								
	Diet and nutrition behavior	✓					✓	✓
	Physical activity	✓					✓	✓
	Quality of life	✓					✓	✓
	Depression	✓					✓	✓
	Smoking	✓						
	Alcohol consumption	✓						
	Drug abuse	✓						
**Anthropometrics & physical examination**								
	Anthropometrics (height, weight, BMI, WC, HC, NC, wrist C)	✓		✓	✓	✓	✓	✓
	Body composition (FM, FFM, LM)	✓		✓	✓	✓	✓	✓
	General physical examination	✓		✓	✓	✓	✓	✓
	Obesity-related comorbidities	✓		✓	✓	✓	✓	✓
**Medical condition**								
	Cardiovascular (ECG, echocardiography)	✓					✓^c^	✓^c^
	Respiratory (Chest X-ray, ABG, PFT, polysomnography)	✓					✓^c^	✓^c^
	Gastrointestinal (endoscopy/barium meal, liver & gallbladder ultrasound)	✓				✓	✓^c^	✓^c^
**Blood & urine assessments**								
	General blood biochemistry (CBC, LFT, LP)	✓				✓	✓	✓
	Glucose homeostasis (FPG, HbA1C)	✓				✓	✓	✓
	Hormonal assessment (TFT, PTH, Insulin)	✓					✓	✓
	Micronutrients (Ca, P, Fe, Cu, Zn)	✓					✓	✓
	Vitamins (D, B12)	✓					✓	✓
	Inflammatory markers (CRP)	✓					✓	✓
	24-hr urine albumin & creatinine	✓					✓^c^	✓^c^
**Surgery**								
	General information (center, date, time, anesthetics)		✓					
	Surgical procedure details		✓					
	Additional/unprecedented procedures		✓					
**Outcomes**								
	30-day surgical complications		✓	✓				
	Long-term complications					✓	✓	✓
	Re-admission^d^					✓	✓	✓
	Reoperation					✓	✓	✓
	EWL%			✓	✓	✓	✓	✓
	Metabolic assessment			✓	✓	✓	✓	✓

^a^WC: waist circumference; HC: hip circumference; NC: neck circumference; Wrist C: wrist circumference; FM: fat mass; FFM: fat-free mass; LM: lean mass; ECG: electrocardiography; ABG: arterial blood gas; PFT: pulmonary function test, CBC: complete blood count:; LFT: liver function test; LP: lipid profile, FBG: fasting plasma glucose; HbA1C: hemoglobin A1C; TFT: thyroid function test; PTH: parathyroid hormone; CRP: C-reactive protein.

^b^ Multimedia Appendix 1 for more information about the online forms.

^c^If necessary.

^d^Including in-patient and out-patient care.

### Data Collection & Quality Control Procedures

An electronic database for precise data collection was designed. Manuals of operations and procedures were also created to minimize technical variability. Data collectors including study investigators and surgeons underwent training and certification with respect to study protocols. Quality control procedures including frequent contacts and visits between the surgeon and clinical center staff help to ensure complete and accurate data collection. Investigators used validated and standardized instruments for objective and subjective measures. When not available, new instruments were created to meet the specific goals of the study (see Multimedia Appendix 1).

A brief summary of each outcome domain and standard forms and measures used to assess each of these domains, as well as the contact points at which they will be administered, are described below.

#### Clinical Endpoints

In order to evaluate the effectiveness of bariatric surgery on obesity and obesity-related comorbidities, all participants are interviewed by a trained physician to complete a standardized clinical history questionnaire. It covers risk factors for cardiovascular disease, hypertension, hyperlipidemia, diabetes, and familial history of non-communicable diseases, smoking habits, drug abuse, and alcohol consumption. A similar postoperative survey is completed as well. The physicians are required to undergo periodic evaluation according to written protocols and control procedures to ensure up-to-date and universal practice.

#### Physical Examination and Anthropometric Measurements

Physical examination aims to look for obesity-related conditions as well as general health status of the individual. Anthropometrics include weight, height, neck, waist, wrist, and hip circumference, measured according to WHO guidelines [29]. Body composition is assessed by a portable bioelectrical impedance analyzer and output data includes body weight (kg), impedance (ohms), fat mass (kg), fat-free mass (kg), total body water (kg), and percent body fat (%) (see Multimedia Appendix 1).

#### Behavioral and Psychosocial Factors

The TOTS investigators have hypothesized that pre-existing psychological and behavioral factors could influence the outcomes after bariatric surgery. These aspects, such as quality of life and depression, will be assessed at baseline and follow-up, and will include questions on preoperative weight loss practices and eating habits (including binge eating and eating beyond satiation), tobacco use (according to US Centers for Disease Control and Prevention) [30], alcohol use, history of psychiatric disorders, and counselor/therapist contact. The depressive symptoms will be assessed using the Persian-language version of Beck Depression Inventory, version 1 [31,32]. Moreover, quality of life will be assessed by the Iranian version of Short Form Health Survey (SF-36) that measures eight health-related concepts, including the physical, mental, and social aspects of health [33]. Physical activity levels are assessed using the Persian-translated long form of International physical activity questionnaire (Persian IPAQ) [34]. The questionnaire measures all three forms of activities including leisure time, job, and household activities in the past week (see Multimedia Appendix 1).

#### Dietary Assessment

Diet plays a central role in the pathophysiology of obesity, as well as obesity treatment. Maintenance of weight loss after bariatric procedures is mainly achieved through changing dietary habits. In order to assess the role of diet on obesity before and after surgical interventions, an expert nutritionist assesses dietary intake of the patients using three consecutive 24-hour recalls, on weekdays (we selected weekdays because weekends do not reflect the usual diet of a patient). Portion sizes of meals are converted to grams by using household measures [35]. Nutrient intakes are calculated according to the US Department of Agriculture and Iranian Food Composition Tables [36,37].

#### Blood and Urine Biochemical Assessment

Blood and urine samples are collected before and after the surgery at 6 and 12 months, and annually thereafter. After 12-14 hours overnight fast, multiple aliquots of blood are drawn for biochemical and future genetic/molecular assessments. A standard 24-h urine collection is advised for all participants (see Multimedia Appendix 1).

#### Genetics and Biomarkers

DNA obtained from consenting participants will be part of an ongoing research effort by our team to identify genes related to human obesity. Blood samples drawn from subjects before and after the surgery are stored for further studies (see Multimedia Appendix 1).

#### Health Care Utilization

Studies addressing cost-effectiveness of bariatric surgery have shown controversial results and have not yet reached a universal conclusion [38-41]. In order to assess the financial burden of obesity in our setting, a dataset is designed to measure and evaluate short- and long-term cost-effectiveness of bariatric surgery pre and postoperatively.

#### Complications Related to Surgery

To document the frequency of complications after these most common techniques, as well as newer techniques in this study, a dataset is designed to describe early (occurring within 30 days of surgery) and long-term complications and factors associated with those events. Early complications known to complicate abdominal surgery include gastrointestinal adverse events (eg, anastomotic leak), thromboembolism, sepsis, and acute kidney injury. Long-term complications include gastrointestinal complications, cardiovascular events, and surgical re-intervention [42].

#### Follow-Up

All participants are scheduled for follow-up visits by a multidisciplinary team (ie, surgeon, obesity specialist, endocrinologist, and dietitian) at 10 days, and at 1, 3, 6, and 12 months, and annually thereafter. A trained nurse will ask about related medical conditions by means of email or over the phone (including cardiovascular, metabolic, pulmonary, renal, musculoskeletal, urologic, reproductive, and gastrointestinal outcomes), and if a related event is noticed, the research investigator will actively seek respective data. In the case of mortality, data will be collected based on the death certificate.

### Approval

This study has been approved by the Human Research Review Committee of the Endocrine Research Center, Shahid Beheshti University of Medical Sciences, No. 2ECRIES 93/03/13.

## Results

This study has recruited 1050 participants as of September 2015 and is ongoing. Mean age of the participants is 37.8 years, mean BMI of 43.7 kg/m^2^, with 76.9% female. Detailed characteristics of the participants including their baseline anthropometrics and prevalence of comorbidities are presented in Table 2.

**Table 2:** Baseline characteristics, anthropometrics, and laboratory values of the participants upon enrollment^a^.

Variables^b^			Total (N=1050)		Female (N=807)		Male (N=243)		*P* value	
Age, year, mean (SD)			37.8 (11.7)		38.6 (11.8)		35.2 (11)		.036	
**Age group, n (%)**									<.001	
	<20		52 (4.2)		39 (4.1)		13 (4.9)			
	20-29		229 (21.8)		162 (19.7)		67 (28.2)			
	30-39		339 (32.8)		251 (31.8)		88 (36.7)			
	40-49		241 (22.8)		197 (24.3)		44 (17.5)			
	50-59		148 (14.7)		123 (15.8)		25 (10.9)			
	60-69		39 (3.5)		34 (4.1)		5 (1.3)			
	≥70		2 (0.2)		1 (0.1)		1 (0.4)			
**Marital status, n (%)**									.014	
	Single/never married		252 (24.1)		169 (25.2)		83 (37.6)			
	Married		668 (61.7)		526 (62.7)		142 (58.4)			
	Divorced		62 (6.2)		52 (6.4)		10 (4.1)			
	Widowed		46 (3.6)		46 (5.7)		0			
**Education, n (%)**									.024	
	No education		10 (1.0)		7 (0.9)		3 (1.2)			
	Primary school		27 (13.8)		23 (15.0)		4 (1.8)			
	College		531 (50.7)		430 (52.2)		101 (45.9)			
	University		448 (34.5)		321 (32.1)		127 (43.1)			
**Employment status, n (%)**									<.001	
	Unemployed		567 (54)		521 (64.6)		46 (18.9)			
	Employed		458 (46)		261 (35.4)		197 (81.1)			
**Smoking status, n (%)**									<.001	
	Never smokers		819 (71.2)		661 (78.9)		158 (44.9)			
	Current smokers		156 (15.4)		89 (11.2)		67 (30)			
	Former smokers		143 (13.4)		80 (9.9)		63 (25.1)			
Hookah use, n (%)			163 (15.5)		91 (11.3)		72 (29.6)		.004	
Alcohol consumption, n (%)			150 (14.3)		86 (10.7)		64 (26.3)		.004	
**Comorbidities, n (%)**										
	Hypothyroidism		186 (17.8)		169 (18.5)		17 (6.5)		<.001	
	Hypertension		174 (17.6)		137 (17.1)		37 (15.3)			
	Dyslipidemia		168 (16.0)		137 (17.0)		31 (12.8)			
	Arthritis		167 (15.9)		144 (17.8)		23 (9.5)		.039	
	Diabetes mellitus		158 (16.6)		127 (17.3)		31 (8.9)			
	Cardiovascular disease		63 (6.0)		47 (5.8)		16 (6.6)			
	Liver Enlargement		350 (92.1)		287 (72.4)		63 (83.5)			
	**Fatty liver**		106 (27.5)		87 (27.4)		19 (27.5)			
		Grade I	134 (35.2)		124 (41.4)		10 (17.1)		
		Grade II	155 (40.7)		125 (41.6)		30 (45.4)		
		Grade III	61 (16.2)		38 (17.0)		23 (33.3)		
	Gallstones		34 (7.1)		30 (7.4)		4 (5.1)			
**Anthropometrics, mean (SD)**										
	Height, cm		163.5 (8.3)		161.2 (6.1)		175.5 (7.8)		<.001	
	Weight, kg (range 74-195)		118.6 (20.7)		114 (17.1)		142.6 (20.9)		<.001	
	BMI, kg/m^2^ (range 32.4-79.3)		43.9 (7.1)		43.8 (5.9)		46.2 (5.5)		.09	
	**BMI group (kg/m^2^), n (%)**								.054
		25-29.9	9 (0.9)		8 (1)		1 (0.4)		
		30-34.9	68 (6.3)		57 (7.4)		11 (4.4)		
		35-39.9	243 (23.2)		197 (24.2)		46 (19.9)		
		40-44.9	332 (32.1)		261 (32.5)		71 (30.7)		
		45-49.9	235 (22.4)		176 (21.5)		59 (25.5)		
		50-54.9	99 (9.3)		67 (8.2)		32 (13)		
		55-59.9	42 (3.7)		24 (3.1)		18 (6.9)		
		60-64.9	13 (1.3)		9 (1.2)		4 (1.7)		
		65-69.9	7 (0.7)		6 (0.8)		1 (0.4)		
		≥70	2 (0.2)		2 (0.3)		0 (0.0)		
	Waist circumference, cm		125.1 (13.9)		122.5 (12.6)		138 (12.6)		<.001	
	Hip circumference, cm		133.6 (12.7)		133.5 (13)		134.4 (11.1)		.065	
	Neck circumference, cm		39.1 (3.9)		37.9 (2.8)		44.7 (3.4)		<.001	
	Wrist circumference, cm		17.9 (1.6)		17.6 (1.4)		19.5 (1.7)		<.001	
	SBP, mmHg		120.1 (16.7)		119.4 (16.6)		124.1 (16.5)			
	DBP, mmHg		75.9 (11.8)		75.3 (11.6)		79 (12.7)			
	Fat mass, %		49.8 (3.9)		50.6 (3.2)		46 (4.7)		<.001	
	Fat mass, kg		58.2 (11.9)		6.9 (10.8)		64.4 (15)		<.001	
	Fat free mass, kg		59 (11.4)		55.5 (7.5)		75.9 (12)		<.001	
**Laboratory values, mean (SD)**										
	FPG, mg/dl		107.9 (34.5)		109.4 (36.5)		99.6 (18.8)		<.001	
	HbA1C, %		5.6 (1)		5.6 (1.1		5.6 (0.7)		.037	
	TG, mg/dl		152.7 (76)		150.3 (70.9)		164.8 (97.7)			
	Cholesterol, mg/dl		192.4 (38)		193.5 (37.4)		186.6 (41.4)		.033	
	HDL, mg/dl		49.6 (10.9)		50.2 (10.8)		46.3 (10.5)		.001	
	LDL, mg/dl		112.5 (32.2)		113 (31.7)		109.6 (35.0)			
	Hemoglobin, g/dl		13.5 (1.5)		13.2 (1.2)		15.2 (1.4)		<.001	
	Hematocrit, %		41.0 (3.9)		40.2 (3.2)		45.7 (3.5)		<.001	
	AST, U/l		23.4 (15.5)		21.8 (12.8)		29.2 (18.6)		.007	
	ALT, U/l		28.7 (21.3)		26.1 (15.5)		41.3 (31.3)		<.001	
	Alkaline phosphatase, IU/l		191.8 (57.7)		191.4 (56.5)		194 (63.8)			

^a^For continuous variables, *t* test was used. For categorical variables, Pearson chi-square test was used.

^b^SBP: systolic blood pressure; DBP: diastolic blood pressure; FPG: fasting plasma glucose; HbA1C: hemoglobin A1C; TG: triglyceride; HDL: high-density lipoprotein, LDL: low-density lipoprotein; AST: aspartate aminotransferase; ALT: alanine aminotransferase

## Discussion

The TOTS is a prospective longitudinal study evaluating many preoperative, operative, and post-operative aspects of bariatric surgery. High fidelity results are assured through the use of standard validated instruments. Results of this study will provide a comprehensive understanding of this growing medical condition and its treatment and will empower clinicians with evidence-based recommendations regarding patient selection and evaluation, surgery options, and follow-up care.

## Appendix

Multimedia Appendix 1 Supplemental information.

## Abbreviations

BMI: Body Mass Index
BPD/BPD-DS: biliopancreatic diversion with or without duodenal switch
EWL: excess weight loss
LAGB: laparoscopic adjustable gastric banding
LSG: laparoscopic sleeve gastrectomy
RYGB: Roux-en-Y gastric bypass
TOTS: Tehran Obesity Treatment Study
