# Supplementary material for: Bariatric Surgery for Morbid Obesity: Tehran Obesity Treatment Study (TOTS) Rationale and Study Design
Source: JMIR Res Protoc. 2016 Jan 20;5(1):e8. doi: 10.2196/resprot.5214 (PMC4740496; doi:10.2196/resprot.5214)
Supplement: Multimedia Appendix 1 [file resprot_v5i1e8_app1.pdf]

Multimedia Appendix 1. Supplemental Information

A.Surgical Procedures 1
I.Roux-en-Y Gastric Bypass (RYGB) 1
I I.Laparoscopic Sleeve Gastrectomy (LSG) 1
I I I.Laparoscopic Adjustable Gastric Banding (LAGB) 2
IV.Gastric plication 2
B.Anthropometric measurements 2
C.Smoking Status 3
D.Blood and urine assessments 3
E.Genetics and biomarkers 4

A. Surgical Procedures

A standard 5-port laparoscopic technique with the bed in the reverse Trendelenburg position is used. At the completion of the procedure, some patients have white silicone drains (SUPA, Iran) placed in the proximity of the gastrojejunostomy (GJ), which are removed at the first postoperative inpatient visit barring any complications. In the interim, patients are asked to record the nature and amount of the output daily. Postoperatively, patients start on oral fluids and are promoted to mobilize. After discharge, patients continue on a liquid diet for 2 weeks followed by a sloppy diet for 4 weeks, before resuming a normal diet. Patients will receive daily multivitamin and mineral supplementations for the first 12 months. All patients undergoing RYGB will receive vitamin B12, vitamin D, and calcium supplements. Iron supplements are given depending on the control blood tests. Acid suppression with a proton pump inhibitor is considered in cases with GI problems. Discontinuation or modification of medical therapy is considered in cases of blood pressure, lipids, or glucose normalization or improvement.. Surgical techniques are as follows:

I . Roux-en-Y Gastric Bypass (RYGB)

Includes an antecolic and antegastric RYGB with an alimentary limb of 100-150 cm and a biliopancreatic limb of 50 cm. A 20–30 cm^3 vertical gastric pouch is created using a 45 or 60-mm stapling device (Endo GIA Auto suture, Covidien, Mansfield, *MA,* USA). A side-to-side GJ is performed using linear stapler. A side-to-side jejuno-jejunostomy is created using a 60-mm

diameter lineal stapler with white loads. Methylene blue or air test is performed to identify possible leaks.

I I . Laparoscopic Sleeve Gastrectomy (LSG)

For the sleeve gastrectomy procedure, 75%-80% of the body and fundus is excised. The gastric tube is created over a 36-F bougie using multiple firings of the stapler. The stapler line is reinforced and imbricated with 2.0 PDS running sutures. A methylene blue or air test is performed to check for leaks and a closed suction drain is placed based on surgeon’s decision.

Mini-gastric bypass

Mini-gastric bypass is a modification of the standard RYGB procedure using a long gastric tube with an antecolic loop GJ. A long gastric tube is created using Endo GIA stapler from the incisura angularis to the angle of His over a 36-F bougie. A loop gastroenterostomy is performed 200 cm distal to the ligament of Trietz with an Endo-GIA stapler. The gastroenterostomy is then reinforced with continuous sutures. A methylene blue or air test is performed to check for leaks and one drain is left in the lesser sac before closure of the wound.

I I I . Laparoscopic Adjustable Gastric Banding (LAGB)

This technique is performed by using the adjustable gastric band with velocity injection port and applier (MIDBAND™, MID, Dardilly, France), applying the “pars flaccida” technique. [1] The band is not secured by fixation to the walls of the stomach and drainage is not routinely used.

IV. Gastric plication

Gastric plication (GP) is a recently introduced bariatric procedure. One of the first descriptions of this procedure came from Iran with the total vertical gastric plication (TVGP). [2] In this operation, the greater curvature of stomach is folded inwards in 3 parallel lines and sewed to reduce the gastric volume without removing any tissue. An intraluminal guide (No. 36) is used to assure the best site for suturing.

B. Anthropometric measurements

Anthropometrics include weight, height, neck, waist, wrist, and hip circumference, measured according to WHO guidelines. [3] Body composition is assessed by the portable bioelectrical impedance analyzer InBody 370 (Biospace, Seoul, Korea). Participants were asked to comply with the following criteria prior to impedance analysis: fasting overnight or for a minimum of 4–5 hours, no exercise for at least 12 hours, no alcohol for at least 24 hours, and balanced hydration. All participants were asked to lie in a supine position for at least 5 minutes prior to examination. After wiping palms and soles with an electrolyte tissue, the patient stands on the foot electrodes and grasps the hand electrodes. Patient characteristics (sex, height, weight, and age) are entered into the instrument. The eight-touch electrodes BIA system measures multiple segmental impedances (right arm, left arm, right leg, left leg and trunk,) with multi-frequency (5, 50, 250 kHz) from tetra-polar electrodes. Resistance to the alternating current flow (500- µA, 50/60 kHz) is measured with the patient standing on the analyzer's platform and interpreted using the "standard" option of the manufacturer's software. Output data includes body weight (in kg), impedance (ohms), fat mass (kg), fat-free mass (FFM, in kg), total body water (TBW, in kg), and percent body fat (%FM).

C. Smoking Status

Smoking status was defined according to CDC definitions for smoking, as shown in

Table 1. Smoking status

Status	Definition
Never Smokers	Adults who have never smoked a cigarette or who smoked fewer than 100 cigarettes in their entire lifetime.
Former Smokers	Adults who have smoked at least 100 cigarettes in their lifetime, but say they currently do not smoke.
Nonsmokers	Adults who currently do not smoke cigarettes, including both former smokers and never smokers.
Current Smokers	Adults who have smoked 100 cigarettes in their lifetime and currently smoke cigarettes every day (daily) or some days (nondaily).

D. Blood and urine assessments

After a 12-14-hour overnight fast, blood samples are drawn in a sitting position between 7-9 a.m. in three aliquots to be stored in -80°C ultra-freezers for biochemical and future genetic/molecular assessments. Blood sample for DNA analysis is stored in an EDTA containing tube. Other samples are then centrifuged within 30 to 45 minutes of collection. The derived serum is separated into at least four 1-ml aliquots; one is sent for all biochemical analyses on the same day, and three others are stored in -80°C ultra-freezers for future studies. Table 2 outlines the blood and urine assessments used in TOTS.

Table 2. Blood and urine assessment in TOTS study

Item	Baseline	1 month	3 month	6 month	12 month	Annually
**Blood assessments**						
CBC	✓			✓	✓	✓
FBG	✓			✓	✓	✓
HbA1c	✓			✓	✓	✓
Fasting insulin level	✓				✓	✓
TG	✓			✓	✓	✓
Total cholesterol	✓			✓	✓	✓
HDL cholesterol	✓			✓	✓	✓
LDL cholesterol	✓			✓	✓	✓
SGOT	✓			✓	✓	✓
SGPT	✓			✓	✓	✓
ALP	✓			✓	✓	✓
Total bilirubin	✓				✓	✓
Serum creatinine	✓				✓	✓
T4	✓				✓	✓
T3RU	✓				✓	✓
TSH	✓				✓	✓
PTH	✓				✓	✓
Iron	✓				✓	✓
Calcium	✓				✓	✓
Phosphor	✓				✓	✓
Copper	✓				✓	✓
Zinc	✓				✓	✓
CRP						
Vitamin D	✓				✓	✓
Vitamin B12	✓				✓	✓
Serum Albumin	✓				✓	✓
Ferritin	✓				✓	✓
TIBC	✓				✓	✓
**Urine Assessments**						
Urine albumin (24-h)	✓				If necessary	If necessary
Urine creatinine (24-h)	✓				If necessary	If necessary
UFC (24-h)	✓				If necessary	If necessary

CBC, Complete blood count; FBG, Fasting blood glucose; HbA1C, Hemoglobin A1C, TG, Triglyceride; SGOT, Serum glutamic-oxaloacetic transaminase; SGPT, Serum glutamic-pyruvic transaminase; ALT, Alkaline phosphatase; T3RU, T3 resin uptake; TSH, Thyroid stimulating hormone; PTH, Parathyroid hormone; TIBC, Total iron binding capacity; UFC, Urinary free cortisol.

E. Genetics and biomarkers

DNA obtained from consenting participants will be part of an ongoing research effort to identify genes related to human obesity. Blood samples drawn from subjects before and after the surgery are stored for further studies. Buffy coats are separated from EDTA-anti coagulated samples and genomic DNA is extracted with the salting-out method [4]. The concentration and purity of the extracted DNA are assayed by Nanodrop 1000 (Thermo Fisher Scientific Inc, Wilmington, DE, USA). The DNA samples are stored at 4°C and back-up DNA samples are stored at -20°C. Tissues are stored in Trizol (Applicam Co, USA) reagent and RNA will be extracted for further analysis.

1 Di Lorenzo N, Furbetta F, Favretti F, et al. Laparoscopic adjustable gastric banding via pars flaccida versus perigastric positioning: technique, complications, and results in 2,549 patients. Surg Endosc. 2010; 24:1519–23

2 Talebpour M, Kalantar Motamedi SM, Talebpour A, Vahidi H. Twelve year experience of laparoscopic gastric plication in morbid obesity: development of the technique and patient outcomes. Annals of Surgical Innovation and Research 2012; 6:7.

3 WHO. Physical status: the use and interpretation of anthropometry. Report of a WHO Expert Committee. World Health Organ Tech Rep Ser 1995; 854: 1–452.

4 Miller SA, Dykes DD, Polesky HF. A simple salting out procedure for extracting DNA from human nucleated cells. Nucleic Acids Res, 1988;16(3):1215.
